# A Liver-Specific Defect of Acyl-CoA Degradation Produces Hyperammonemia, Hypoglycemia and a Distinct Hepatic Acyl-CoA Pattern

**DOI:** 10.1371/journal.pone.0060581

**Published:** 2013-07-05

**Authors:** Nicolas Gauthier, Jiang Wei Wu, Shu Pei Wang, Pierre Allard, Orval A. Mamer, Lawrence Sweetman, Ann B. Moser, Lisa Kratz, Fernando Alvarez, Yves Robitaille, François Lépine, Grant A. Mitchell

**Affiliations:** 1 Centre de Recherche and Département de Pédiatrie (NG, JWW, SPW, PA, FA, GAM) or Département de Pathologie (YR), CHU Sainte-Justine, Université de Montréal, Montréal, Québec, Canada; 2 Département de Biochimie, Université de Montréal, Montréal, Québec, Canada; 3 Goodman Cancer Research Centre, McGill University, Montréal, Québec, Canada; 4 Institute of Metabolic Disease, Baylor Research Institute, Dallas, Texas, United States of America; 5 The Hugo W Moser Research Institute, The Kennedy-Krieger Institute, Johns Hopkins School of Medicine, Baltimore, Maryland, United States of America; 6 INRS-Institut Armand-Frappier, Laval, Québec, Canada; National Institute of Agronomic Research, France

## Abstract

Most conditions detected by expanded newborn screening result from deficiency of one of the enzymes that degrade acyl-coenzyme A (CoA) esters in mitochondria. The role of acyl-CoAs in the pathophysiology of these disorders is poorly understood, in part because CoA esters are intracellular and samples are not generally available from human patients. We created a mouse model of one such condition, deficiency of 3-hydroxy-3-methylglutaryl-CoA lyase (HL), in liver (HLLKO mice). HL catalyses a reaction of ketone body synthesis and of leucine degradation. Chronic HL deficiency and acute crises each produced distinct abnormal liver acyl-CoA patterns, which would not be predictable from levels of urine organic acids and plasma acylcarnitines. In HLLKO hepatocytes, ketogenesis was undetectable. Carboxylation of [2-^14^C] pyruvate diminished following incubation of HLLKO hepatocytes with the leucine metabolite 2-ketoisocaproate (KIC). HLLKO mice also had suppression of the normal hyperglycemic response to a systemic pyruvate load, a measure of gluconeogenesis. Hyperammonemia and hypoglycemia, cardinal features of many inborn errors of acyl-CoA metabolism, occurred spontaneously in some HLLKO mice and were inducible by administering KIC. KIC loading also increased levels of several leucine-related acyl-CoAs and reduced acetyl-CoA levels. Ultrastructurally, hepatocyte mitochondria of KIC-treated HLLKO mice show marked swelling. KIC-induced hyperammonemia improved following administration of carglumate (N-carbamyl-L-glutamic acid), which substitutes for the product of an acetyl-CoA-dependent reaction essential for urea cycle function, demonstrating an acyl-CoA-related mechanism for this complication.

## Introduction

Much of the intermediary metabolism of proteins and fatty acids proceeds by the sequential oxidation of acyl-CoAs, and most of the conditions detected by expanded newborn blood screening are caused by deficiency of one of the many intramitochondrial enzymes that normally degrade acyl-CoAs. Examples include methylmalonic, propionic, isovaleric and 3-hydroxy-3-methylglutaric acidemias. Each condition can present a complex and distinct array of manifestations. However, many of these conditions have in common a state of chronic illness with acute and potentially fatal episodes of lethargy, coma, hypoglycemia and hyperammonemia. Key acyl-CoAs, particularly acetyl-CoA, are critical for normal function of the urea cycle [1], the Krebs cycle [2] and for acetylation and regulation of many metabolites and proteins [3].

The potential importance of acyl-CoA esters in inherited metabolic diseases was predicted over 20 years ago [4], [5]. However, to directly explore whether abnormal patterns of acyl-CoAs occur in inborn errors of acyl-CoA metabolism and if so, whether they have pathological importance, requires a sensitive, general assay of CoA esters and a suitable biological model with findings similar to those of human patients. Mass spectrometry-based assays, developed for specific acyl-CoAs [6], [7], could be expanded to a general assay of hepatic acyl-CoAs. Seminal studies in normal rodent hepatocytes showed rapid transient shifts in acyl-CoA pools following loading with precursor compounds [8], [9]. Pharmacological studies in mice show that such studies are feasible, including treatment with CoA synthesis inhibitors [10] and xenobiotic organic acids that form acyl-CoAs [11]. CoA esters are intracellular, and their measurement requires tissue samples. A small number of case reports in human patients have directly examined acyl-CoA levels in hereditary diseases of acyl-CoA metabolism [12], [13]. An animal model would provide tissue samples in a controlled fashion.

**Figure 1:**
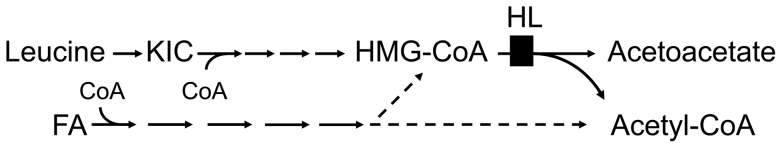
Two metabolic pathways lead to mitochondrial HMG-CoA. Leucine and its metabolite KIC are degraded entirely via HMG-CoA. In hepatic ketogenesis from fatty acid oxidation, a variable fraction of the flux is directed towards HMG-CoA, in response to physiological demands for ketone bodies.

3-hydroxy-3-methylglutaryl-CoA (HMG-CoA) lyase (HL, gene *Hmgcl*, E.C. 4.1.3.4) [14], [15] catalyzes the final step of both leucine degradation and of hepatic ketogenesis from fatty acids (Figure 1). HL-deficient patients appear normal at birth but are subject to severe episodes of hypoglycemic coma. Avoidance of fasting and of very high fat intake, plus moderate limitation of protein intake, helps to prevent such crises. To study whether abnormal liver acyl-CoA metabolism could produce the classical signs of inborn errors of metabolism, and if so, how, we created mice with liver-specific deficiency of HL.

## Materials and Methods

### Chemicals

3-hydroxybutyrate dehydrogenase and bovine serum albumin were from Roche Diagnostics (Indianapolis, IN). Type 1 collagenase was from Worthington (Lakewood, NJ). The [2-^14^C] pyruvate was from Perkin-Elmer (Boston, MA). Other chemicals were from Sigma-Aldrich (Saint Louis, MO) except if noted otherwise.

### Production of liver-specific HL-deficient mice

Construction of the gene targeting vector and targeting in embryonal stem cells are described in Supplemental Information. Targeted embryonal stem cell clones were microinjected into C57BL/6J blastocysts and transferred to pseudopregnant recipients. We obtained 4 chimeras from one clone and 6 from the other. Chimeras were bred to C57BL/6J mice. Agouti offspring were genotyped to identify heterozygotes (HL^+/L^). In order to obtain the excision in liver of HL exon 2, which is catalytically essential [16], HL heterozygotes (HL^+/L^) were bred to Alb-Cre mice (B6.Cg-Tg (Alb-cre) 21 Mgn/J, 003574, the Jackson Laboratory, Bar Harbor, ME). Alb-Cre mice express Cre recombinase from the hepatocyte-specific albumin promoter. HL^+/L^Cre^+^ mice were crossed to obtain Cre transgenic HL^L/L^ homozygotes (HL^L/L^Cre^+^; henceforth designated HL liver knockout (HLLKO) mice). Genotyping of normal, targeted and excised *Hmgcl* alleles and of the Cre transgene is described in Supporting Information.

### Mouse breeding and care

Mice described in this article were obtained by transferring the targeted HL allele to a *C57BL/6J* background for eight generations, then introducing the Alb-Cre allele and interbreeding. Mice were housed with a 12-h light-dark cycle, with the light phase from 6:00 AM to 6:00 PM. They were fed Teklad Mouse Breeder Diet (W) 8626 (Harlan Laboratories, Inc., Madison, WI) containing 20.6% protein and 1.86% leucine (w/w), and 10.4% fat.

At 3–4 weeks of age, mice were genotyped and the results compared with the expected Mendelian ratios. Cre^+^ HL^+/+^, Cre^−^ HL^+/L^ and Cre^−^ HL^L/L^ littermates served as controls for HLLKO mice. After the discovery of high mortality on a normal diet as described in Results, HLLKO mice and controls were weaned to a modified isocaloric diet containing 6.1% protein (0.55% leucine) and 5.5% fat (TD 90016, Harlan Laboratories, Inc., Madison, WI). Furthermore, glucose was added to the drinking water (10% w/v). All protocols were approved by the CHU Sainte-Justine Animal Care Committee.

### Southern, Northern and Western blotting

**Figure 2:**
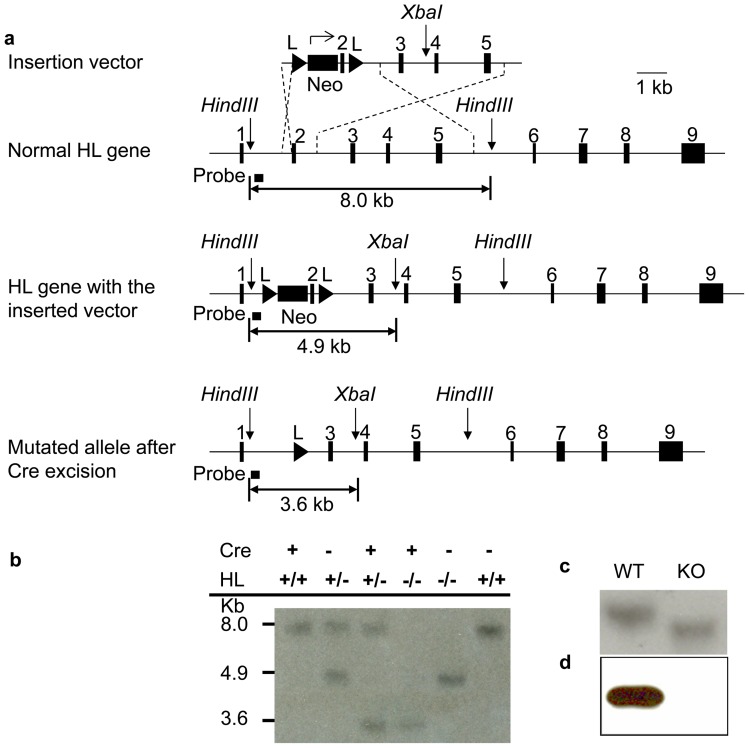
Successful targeting of the HL gene, liver-specific gene excision and liver HL deficiency. (a) The upper diagram shows the introduction of a neomycin construct between exons 1 and 2. Two LoxP sequences are inserted, 5′ to the neomycin construct and after exon 2. The lower panel demonstrates the sizes of diagnostic restriction fragments in the normal gene, the floxed targeted allele and the allele following Cre-mediated excision. Fragments are generated by *Hin*dIII-*Xba*I double digestion. (b) Genomic Southern blot to detect targeting of the HL locus in mouse hepatocytes. Genotypes and molecular weights are indicated. (c) Northern blot of liver RNA using the HL cDNA probe. Each lane contains 10 μg of liver RNA. (d) Western blot analysis of liver mitochondrial HL. In each lane, 10 μg of protein is present.

Southern blots were performed as described [17] with the following modifications. *Hin*dIII – *Xba*I double digests were performed on 5 µg of genomic DNA. Probes used were an amplified fragment spanning intron 1 residues 799–1130 nt upstream of the acceptor splice site (Figure 2a). Northern [18] and Western analyses [19] were as described.

### HL assay

HL activity was measured spectrophotometrically as described [17].

### Hepatocyte isolation

Hepatocyte isolation was based on Seglen [20]. Briefly, following anesthesia with sodium pentobarbital, 65 mg/kg, the portal vein was canulated with a 24 gage 3/4 inch catheter and the vena cava was sectioned. The liver was perfused at 7 mL/min for 5 min with washing buffer (137 mM NaCl, 2.7 mM KCl, 282 mM Na_2_HPO_4_ and 10 mM HEPES) then for 8 min (or sufficient digestion) with digestion buffer, which had the same composition as washing buffer plus 0.33 mg/mL collagenase (Type 1 Worthington Lakewood, NJ) and 1 mg/mL CaCl_2_. The liver was then transferred to a Petri dish. The capsule was disrupted. Hepatocytes were suspended in L-15 buffer (Invitrogen, Grand Island, NY) with 0.2% bovine serum albumin, passed through a 74 μm nylon membrane, then centrifuged three times at 50 g for 2 min at 4°C. Cell viability of ≥90% was required for further testing, as evaluated by exclusion of trypan blue (2 g/L in phosphate buffered saline).

### Ketogenesis

Hepatocytes were suspended at a concentration of 10^6^ cells/mL in Krebs buffer and incubated for 30 min at 37°C in the presence or absence of 2 mM octanoate. The reaction was stopped by perchloric acid precipitation (200 μL, 3 M) followed by neutralization with 400 μl of 2 M KOH/0.3 M MOPS. The production of acetoacetate from octanoate was assayed spectrophotometrically, as in the HL assay above.

### ^14^CO_2_ production from [2-^14^C] pyruvate

This was assayed as described [21]. Briefly, liver mitochondria were isolated as described [21], then incubated in the presence or absence of KIC, 4 mM in 3 mL of sucrose (250 mM), glucose (20 mM), 3-hydroxybutyrate (1 mM), succinate (20 μM), KHCO_3_ (13 mM), MgCl_2_ (11 mM), K_2_HPO_4_ (2 mM), EGTA (0.5 mM) and HEPES (3 mM) pH 7.2, for 30 min at 30°C, then incubated with [2-^14^C] pyruvate (8 Ci/mol; final total pyruvate concentration, 52 μM), ATP (1 mM) and hexokinase (1 U/mL) for 30 min at 30°C in a 25 mL Erlenmeyer flask with a center well (Kontes glassware, Vineland, NJ) in a shaking water bath. The reaction was stopped with 0.3 mL HClO_4_ 20%, by injection of 0.3 mL of methylbenzonium hydroxide into the well to trap ^14^CO_2_. Scintillation counting was performed.

### KIC challenge testing

This test was performed by injecting KIC, 2 mg/g body weight (15.4 µmol/g) intraperitoneally to mice.

### Intraperitoneal pyruvate tolerance test

For this test of gluconeogenesis, 4–5 month-old control and HLLKO mice were fasted until blood glucose was <7 mmol/L. Sodium pyruvate, 2 g/kg in saline was injected i.p. The rise of plasma glucose was followed as described [10].

### KIC-induced hyperammonemia and carglumate administration

Two groups of HLLKO mice and controls were studied. In each, intraperitoneal injection of 2 mg/g of KIC was performed, then food was removed and pure water was substituted for 10% glucose. Behaviour and glycemia were assessed at 90 min, 180 min, then every 60 min. If glycemia was <6 mmol/L, 500 µL of 10% glucose (37°C) were injected intraperitoneally. When lethargy or glycemia <6 mmol/L occurred, plasma ammonia was assayed. In the first group, when plasma ammonia exceeded 200 µmol/L, carglumate (N-carbamyl-L-glutamic acid, carglumic acid, Sigma-Aldrich, St Louis, MO, 0.5 mg/g, dissolved in water) was administered by gavage 60 min after the blood draw. Plasma ammonia was measured again 2 h after carglumate gavage. For each HLLKO mouse, a paired control was treated in the same fashion.

The second group of HLLKO and control mice were treated similarly except that after the first plasma ammonia >200 µmol/L, they received a gavage of water only. Plasma ammonia was measured 2 h later. At that point, mice with plasma ammonia >200 µmol/L received carglumate and were followed as above.

### Sources and synthesis of short-chain acyl-CoA standards

Purified acyl-CoAs were commercially-available for several intermediates of leucine degradation (isovaleryl-CoA, 3-methylcrotonyl-CoA, 3-hydroxy-3-methylglutaryl-CoA), the Krebs cycle (acetyl-CoA, succinyl-CoA) and last stages of fatty acid oxidation (acetoacetyl-CoA, butyryl-CoA), all from Sigma-Aldrich (Saint Louis, MO). A deuterated acetyl-CoA was synthesized as follows. Coenzyme A (25 mg, 0.0325 mmol) was added to 2 mL of ice-cold water. D_3_-acetic anhydride, 20 µL (0.23 mmol, CDN Isotopes Inc, Pointe-Claire, Qc) was added with shaking and vortexing. The solution had a pH of approximately 5 at that step. After 20 min at 0°C, another 20 µL of D_3_-acetic anhydride were added, vortexed and the mixture was kept on ice for 30 min. The pH was lowered to ∼2 by addition of 10 µL of formic acid. Analysis was by direct infusion liquid chromatography-quadrupole time of flight (LC-QTOF).

### Sample preparation for acyl-CoA profiling

Animals were killed by exposure to CO_2_ followed by cervical dislocation. The liver was rapidly excised, frozen in liquid nitrogen and then powdered under liquid nitrogen. For each analysis, precisely-measured amounts (between 0.1 to 0.2 g) of powdered tissue were spiked to a final concentration of 20 ppm in a final volume of 100 μL with the [D_3_]acetyl-CoA standard, then homogenized in 2 mL ice-cold 10% trichloroacetic acid with 2 mM DTT using a Polytron (Kinematica Inc, Bohemia, NY). The tubes were vortexed for 5 sec and centrifuged at 4°C for 5 min at 13,000 g. The supernatants were then applied to a 3 cc Oasis HLB solid-phase extraction column (Waters, Milford, MA, USA) preconditioned with 2 mL of methanol and 2 mL of water. The column was then washed with 2 mL of 2 mM DTT in water and eluted with 2 mL of 2 mM DTT in methanol. The eluate was evaporated under a stream of nitrogen, reconstituted in 100 µL of 2 mM DTT in water. 20 µL served for high performance liquid chromatography coupled to tandem mass spectrometry (HPLC/MS/MS) analysis.

### HPLC/MS/MS assay of short chain acyl-CoAs

The HPLC/MS/MS system consists of a 2795 Waters HPLC coupled to a Micromass Quattro Premier XE (Milford, MA, USA). The column was a 150×3 mm Gemini-NX C18 (5 microns) from Phenomenex (Torrance, CA). Eluent A was 2 mM ammonium acetate in water and eluent B was 2 mM ammonium acetate in acetonitrile. The gradient was 100% A for 5 min, going to 50% B after 30 min, then to 100% B after 31 min, maintained at this composition until 36 min, returning to the initial composition at 37 min and stabilized until 42 min. Flow rate was 0.4 mL/min. The MS was operated in negative ionization electrospray with the following settings: desolvation gas 100 L/Hr; cone gas 10 L/Hr; capillary voltage 2.5 kV; source temperature 120°C; and cone voltage 20 V.

The mass spectrometric data were obtained in multiple reaction monitoring acquisition mode for nine short chain acyl-CoA species using the following transitions (m/z) and collision energies: free CoA (382.5>685.9, 17 V), succinyl-CoA (432.5>685.7, 15 V), isovaleryl-CoA (424.5>769.9, 18 V), HMG-CoA (454.5>382.5, 15 V), acetoacetyl-CoA (424.6>382.4, 11 V), butyryl-CoA (417.7>755.7, 17 V), methylcrotonyl-CoA (423.7>685.7, 20 V), acetyl-CoA (403.6>728, 15 V) and the internal standard [D_3_]acetyl-CoA (404.6>730.9, 15 V). The parent and daughter ions and the collision energy used for each acyl-CoA multiple reaction monitoring were determined using pure samples. Standard curves were constructed for each acyl-CoA using pure molecules. Standard curves were spiked with the internal standard [D_3_]acetyl-CoA to compare the relative response factor between each molecule and the standard for the quantification of those short-chain acyl-CoAs in the mouse liver sample.

### MS determination of unidentified acyl-CoAs

To identify unknown acyl-CoA species, analyses were performed on a 6224 TOF MS coupled to a 1260 Infinity HPLC system, both from Agilent Technologies Inc. Ionization was performed in negative mode on a dual spray ESI source and mass spectra were acquired from m/z 100 to 3200. Samples were diluted to 50 µL, then 2 µL aliquots were injected into the LC-MS system. The chromatographic column was an XBridge C18, 3.5 µm, 4.6×50 mm from Waters. Elution was performed under a two step gradient using acetonitrile and 10 mM ammonium acetate as mobile phases. Deprotonated species were taken into account for accurate mass calculation.

### Hepatic histology and ultrastructure

Liver fragments in buffered formalin were paraffin-embedded for hematoxylin and eosin staining and fixed in OCT for Oil Red O staining. Other fragments were fixed in 0.1 M phosphate-buffered 3% glutaraldehyde (pH 7.4) for 2 hours at room temperature, stored in 0.1 M phosphate buffer (pH 7.4), then postfixed in 1% OsO4 for one hour at 4°C, dehydrated in ethanol, and embedded in Epon for electron microscopy.

## Results

### HLLKO hepatocytes show complete *Hmgcl* gene excision and absence of HL protein and activity

The targeted, excised *Hmgcl* allele is identified by a 3.6 Kb *HindIII-XbaI* fragment (Figure 2a). This was present in genomic Southern blots of whole liver DNA from HLLKO mice (not shown). A faint 4.9 Kb fragment was also seen in whole liver DNA, consistent with lack of excision in liver cells other than hepatocytes [22]. DNA from isolated HLLKO hepatocytes showed only the 3.6 Kb fragment, consistent with complete excision (Figure 2b). Northern analysis of HLLKO liver extract (Figure 2c) showed an abnormal short HL mRNA, as expected for deletion of exon 2 (60 nt). By immunoblotting, HL protein was undetectable in hepatocyte mitochondria from HLLKO mice but was clearly present in control hepatocytes (Figure 2d). HL activity in HLLKO liver homogenates was 0.32±0.24 nmol acetoacetate/min/mg protein (n = 3), at the lower limit of detection and about 100-fold less than in control livers (34.61±9.41 nmol acetoacetate/min/mg protein, n = 3; p<0.05).

### Spontaneous fatal episodes of lethargy, hypoglycemia and hyperammonemia in HLLKO mice after weaning

**Figure 3:**
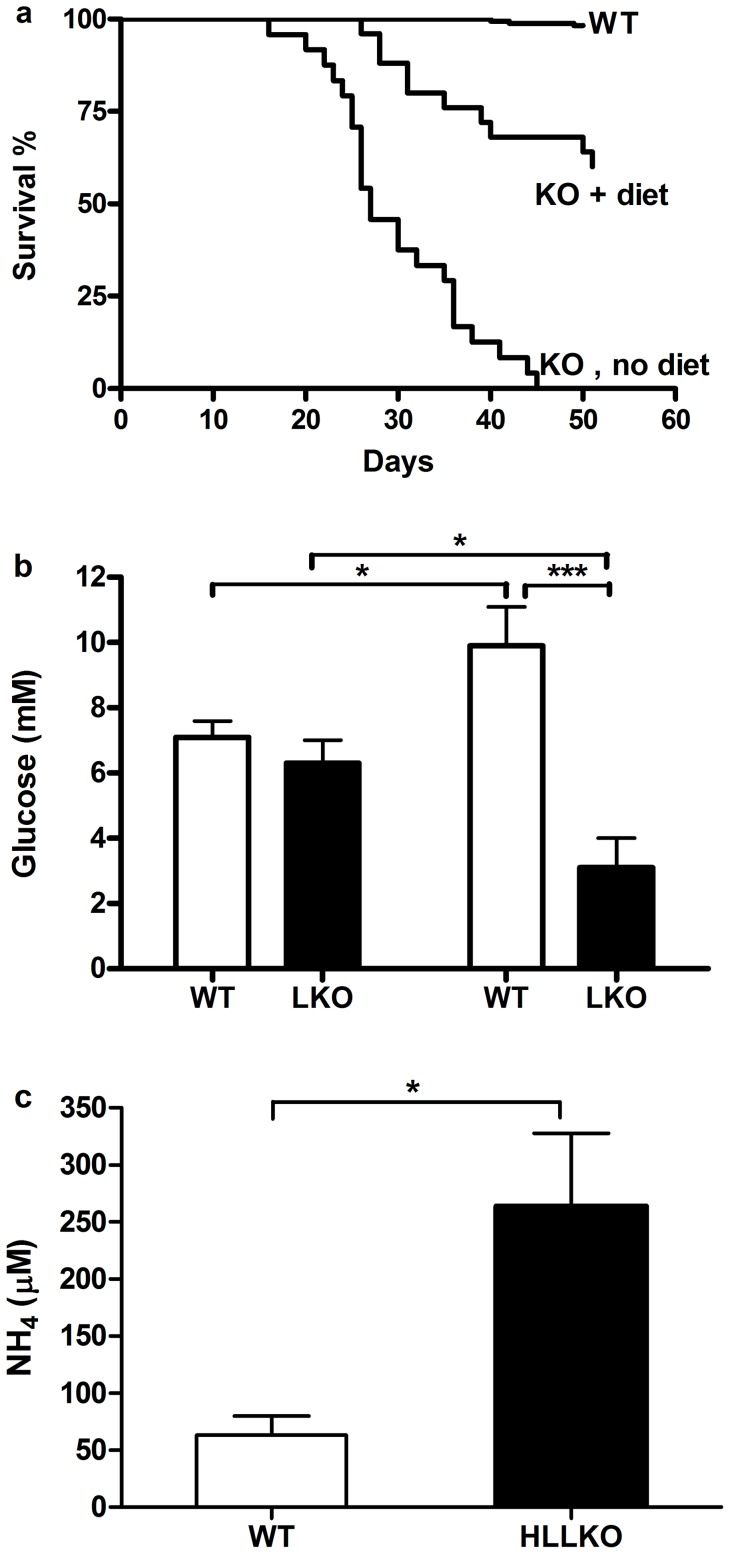
Fatal hypoglycemic, hyperammonemic coma in HLLKO mice. (a) Survival curves of the HLLKO mice with and without the modified diet are compared to a control group. HLLKO without diet, n = 24; HLLKO with diet, n = 25; controls, n = 25. (b and c) KIC injection causes hypoglycemic, hyperammonemic episodes in HLLKO mice. Mice received an injection of KIC, 2 mg/g and were tested at the onset of crises, which occurred 1–4 hours later. Mice were paired with for age- and sex-matched controls and were 3-8 months old. (b) Blood glucose levels of HLLKO and control mice before and during crises, n = 7. (c) Blood NH_4_ levels of HLLKO and control mice during crises., n = 5. Unfilled bars, controls; filled, HLLKO.

Under standard animal room diet conditions, 24/24 (100%) of HLLKO mice died between 3 and 5 weeks of age (Figure 3a). Genotyping at 1 month of age revealed the expected Mendelian ratios, suggesting that HLLKO mice had normal viability during pregnancy and suckling. HLLKO mice appeared normal until 1–3 h before death. For each of these spontaneous episodes, henceforth termed “crises” that were detected before death, progressive lethargy occurred over 1–4 hours and, when measured, hypoglycemia (1.9±0.3 mM (mean ± SEM); range <0.6 to 3.6 mM; n = 13; normal, 7.3±0.4 mM, n = 10) and hyperammonemia (455±139 μM; 300 to 732 μM; n = 4; normal, 94±17 μM, n = 5) were present.

### Intakes of leucine or leucine metabolites strongly influence the clinical course of HLLKO mice

The occurrence of crises in HLLKO mice was diminished by feeding them a chow with low protein content (30% that of normal chow) plus adding 10% glucose to their drinking water. With these measures, >60% of HLLKO mice survived until needed for experiments (Figure 3a). This diet was used in all further experiments.

Conversely, injection of KIC, the transamination product of leucine (2.0 mg/g body mass) provoked fatal crises. Lethargy and unresponsiveness to external stimulation were detectable 1–3 hours after injection (n = 7), and were associated with hypoglycemia (3.1±0.9 mM, compared to 9.9±1.2 mM in controls, n = 7, p<0.001, Figure 3b). Plasma ammonia levels were normal in nonstressed HLLKO mice (not shown), but elevated in HLLKO mice in crises (263±64 μM; KIC-injected normal controls, 63±17 μM, n = 5, p<0.05; Figure 3c).

### HL deficiency confined to liver produces metabolite patterns in urine and plasma like those of HL-deficient humans

Urine organic acids and plasma acylcarnitines were measured in samples from HLLKO mice and controls under non-stressed conditions, following KIC injection, and in samples available from spontaneous crises in HLLKO mice. Urine organic acids showed high variablity within groups, but some patterns emerged. Under stable conditions, HLLKO mice had elevated levels of leucine-related metabolites (Table S1). During spontaneous episodes, and following KIC injection, the mean values of most urine leucine metabolites were 4- to 22-fold higher than in nonstressed mice, and 3-hydroxyisovaleric acid was 250-fold greater. In spontaneous crises of HLLKO mice, mean levels of Krebs cycle and fatty acid derivatives were also higher than in controls.

Plasma acylcarnitines under nonstressed conditions were similar in HLLKO and control mice (Table S2). After KIC injection, both HLLKO and control mice showed increases in C5-hydroxylcarnitine. In spontaneous crises, HLLKO mice showed elevated C5-hydroxylcarnitine, and also significant increases in FA-derived acylcarnitines and decreased C4- and C3-acylcarnitines.

### Acyl-CoA patterns of HLLKO liver show widespread elevations of leucine-related acyl-CoAs and specific changes following KIC loading

Each of the acyl-CoA standards was clearly resolved by HPLC. Each of the corresponding endogenous acyl-CoAs was identified in normal liver and easily quantifiable in a single run. After collision-induced dissociation, each acyl-CoA generated an ion at 382 m/z, representing the free CoA moiety cleaved at the thioester bond.

**Figure 4:**
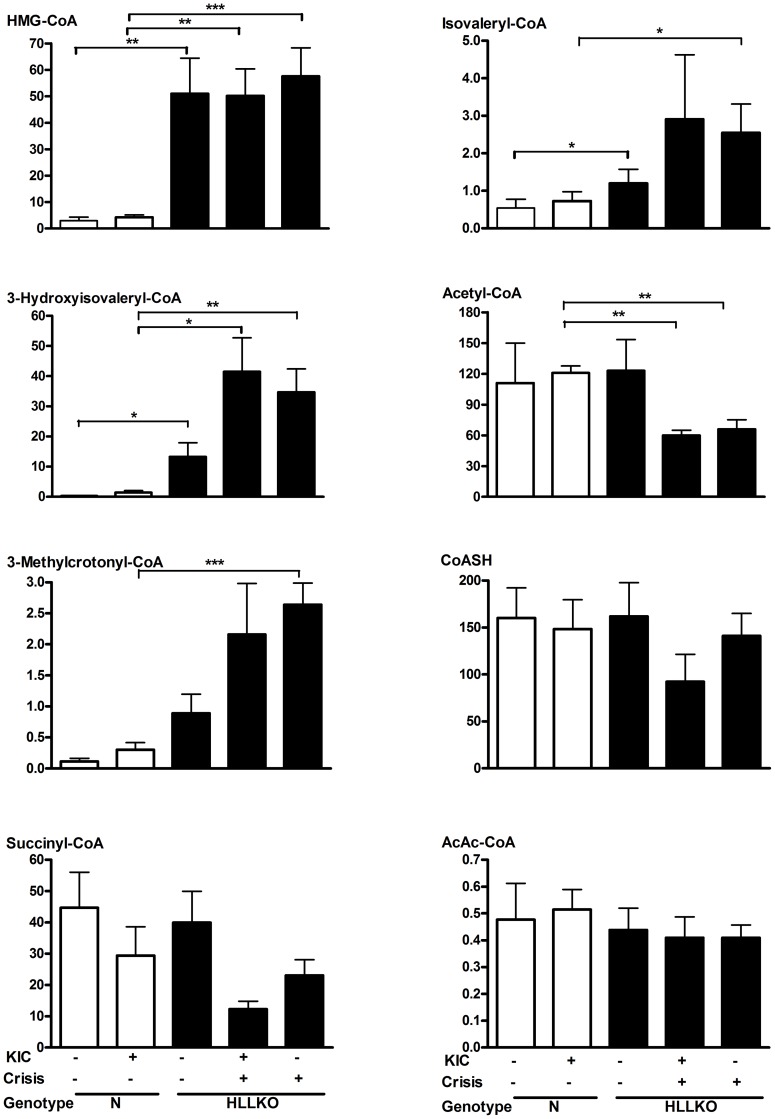
Liver short-chain acyl-CoA patterns in normal and HLLKO liver. Stable and KIC-injected mice (n = 5) were paired with age- and sex-matched controls. Four mice in spontaneous crises were also studied. Acyl-CoA levels are shown as nmol/g liver (wet weight). Unfilled bars, controls; filled, HLLKO.

In HLLKO liver from stable nonstressed mice, the main finding was a marked increase of leucine-related acyl-CoAs, including HMG-CoA (Figure 4). In KIC-induced crises, HMG-CoA levels in HLLKO liver were similar to those of non-stressed HLLKO liver, but the levels of other leucine-related acyl-CoAs changed dramatically. Importantly, during spontaneous or KIC-induced crises in HLLKO liver, acetyl-CoA level, which was normal under non-stressed conditions, was two-fold less than normal (p<0.01). Mean succinyl-CoA level was lower under stressed conditions but this did not reach statistical significance. Mean levels of butyryl-CoA (not shown) varied from 1 to 4 nmol/g wet weight in normal and HLLKO livers and were not statistically different under any condition.

**Figure 5:**
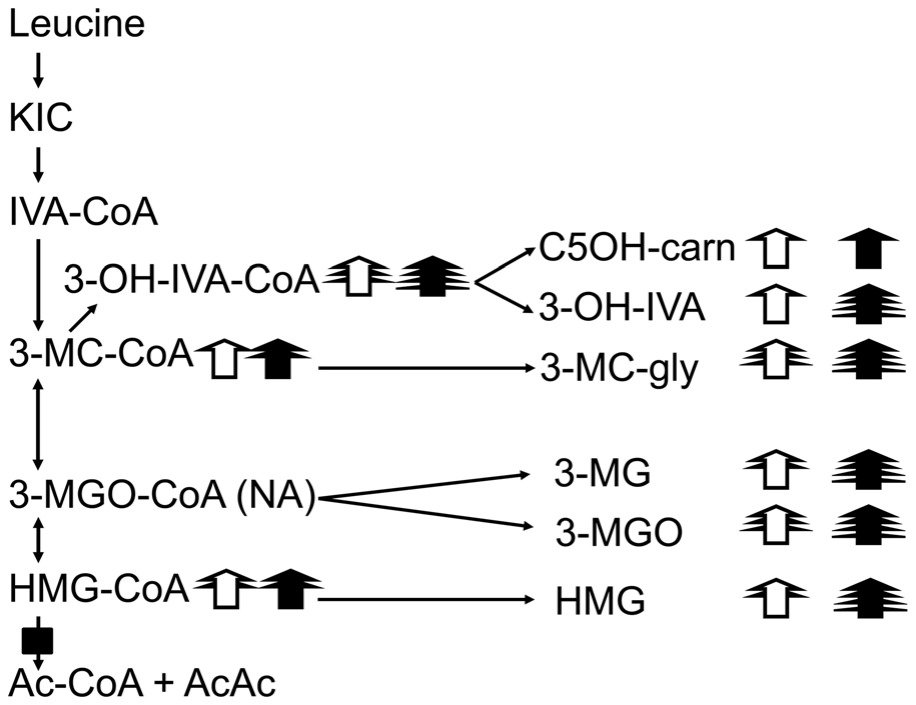
Patterns of leucine-related liver acyl-CoAs and urine and plasma metabolites. The intermediates of leucine degradation are shown on the left. The metabolic block at HL is shown. Related urine and plasma metabolites are shown on the right. The number of arrowheads reflects the magnitude of increase with respect to normal, nonstressed control mice for HLLKO mice under normal conditions (unfilled arrows) and during decompensations filled arrows: 1 arrowhead, 3 to <9-fold increase; 2, 9-<27X; 3, 27-<81X; 4, >81X. Abbreviations, IVA-CoA, isovaleryl-CoA; 3-MC-CoA: 3-methylcrotonyl-CoA; 3-MGO-CoA, 3-methylglutaconyl-CoA; 3-OH-IVA-CoA, 3-hydroxyisovaleryl-CoA; C5OH-carn: C5 hydroxycarnitine; 3-OH-IVA, 3-hydroxyisovaleric acid; 3-MC-gly, 3-methylcrotonylglycine; 3-MGO, 3-methylglutaconic acid; 3-MG, 3-methylglutaric acid. NA, not available (see text).

The identities of two other acyl-CoAs were studied with LC/TOF-MS. A major species on HPLC-MS/MS had a molecular weight of 867 Da. Biologically-occurring candidates with this molecular mass include succinyl-, methylmalonyl-, 2-methyl-3-hydroxybutyryl- and 3-hydroxyisovaleryl-CoAs. On liquid chromatography, pure samples of succinyl- and methylmalonyl-CoAs each had different retention times than this peak. The compound had an exact mass of 867.1676 Da on TOF-MS analysis which is compatible with either 3-hydroxyisovaleryl-CoA or 2-methyl-3-hydroxybutyryl-CoA. 2-methyl-3-hydroxybutyryl-CoA is an isoleucine intermediate that is not commercially available and is not predicted to increase following KIC loading. In contrast, 3-hydroxyisovaleryl-CoA is a byproduct of leucine degradation (Figure 5) and 3-hydroxyisovaleric acid is elevated in the urine of HL-deficient patients [14] and HLLKO mice (Table S1). We therefore designate the observed acyl-CoA peak as 3-hydroxyisovaleryl-CoA. A very small amount of 3-methylglutaconyl-CoA was also detected with LC/TOF-MS (893.14400 Da) although no corresponding peak was found on HPLC-MS/MS analysis.

### HLLKO liver shows lack of ketogenesis, low CO_2_ production from pyruvate and lack of gluconeogenesis

**Figure 6:**
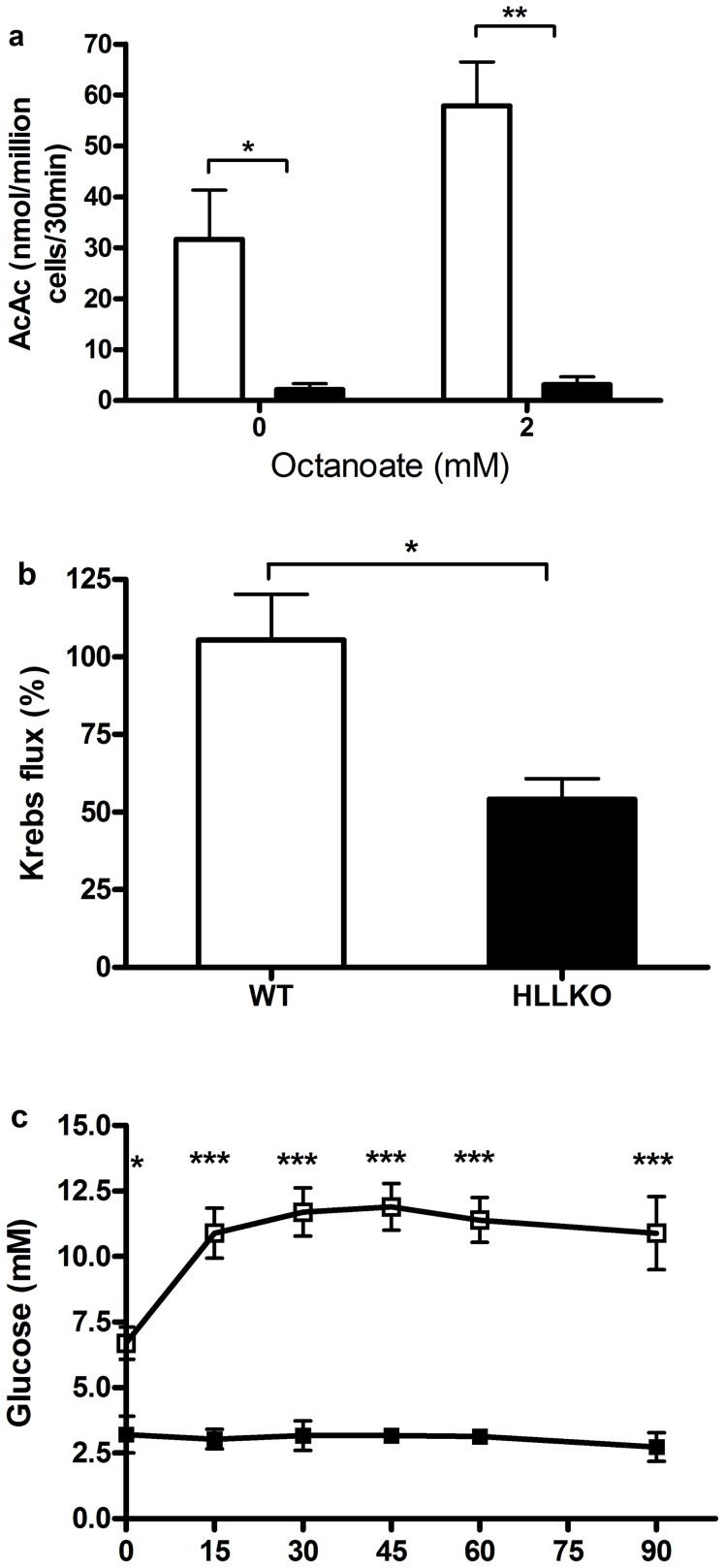
HLLKO mice show suppression of ketogenesis, Krebs cycle flux and gluconeogenesis. (a) Ketogenesis from octanoate is undetectable in HLLKO hepatocytes but is abundant in control hepatocytes. Hepatocytes (10^6^/mL) were suspended in Krebs buffer with the indicated concentration of octanoate, then incubated for 30 min at 37°C. The reaction was stopped and acetoacetate was measured. Three experiments were performed for each genotype. Unfilled bars, controls; filled, HLLKO. (b) Pre-incubation with KIC reduces Krebs cycle flux in HLLKO hepatic mitochondria. Krebs cycle flux is measured as ^14^CO_2_ production from [2-^14^C]pyruvate in isolated liver mitochondria. The effect of KIC incubation was expressed as the fraction of ^14^CO_2_ generation in KIC-incubated mitochondria compared to mitochondria incubated without KIC. Controls, n = 5; HLLKO, n = 3. Unfilled bars, controls; filled, HLLKO. (c) HLLKO mice show no detectable gluconeogenesis following pyruvate administration. 4-month-old mice were fasted until blood glucose was <6 mmol/L (11–17 hours), then received injections of 2 g/kg pyruvate i.p. Controls, n = 8; HLLKO, n = 3.

In isolated hepatocytes from control mice, acetoacetate production was easily detectable under baseline conditions and was increased during incubation with octanoate (Figure 6a). In contrast, in HLLKO hepatocytes, acetoacetate production was undetectable in either condition. ^14^CO_2_ production from [2-^14^C] pyruvate, a measure of Krebs cycle flux, was reduced in HLLKO mitochondria by incubation with KIC, whereas control liver mitochondria showed no change in response to KIC (Figure 6b). To measure gluconeogenesis *in vivo*, intraperitoneal pyruvate loading was performed. The expected rapid increase of blood glucose was observed in controls, but no detectable increase in blood glucose occurred in HLLKO mice (Figure 6c).

### Hyperammonemia in HLLKO mice improves following carglumate treatment

**Figure 7:**
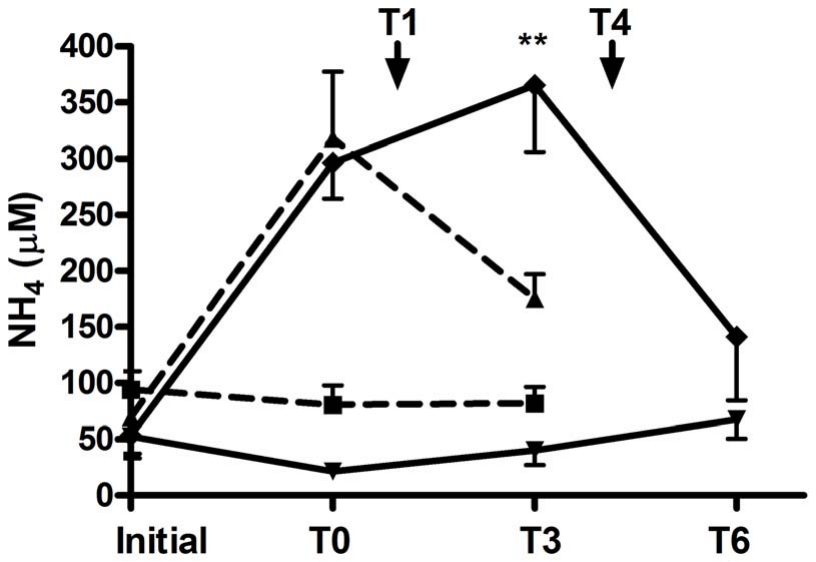
Carglumate treatment corrects hyperammonemia in HLLKO mice. T0 indicates the time at which hyperammonemia <200 µmol/L was first demonstrated (see text). T1 and T4 indicate the times of administration, 1 h and 4 h respectively after T0, for early or late treatment with carglumate. Late treated mice and their controls received a sham gavage at 1 h. Each group contained 5 mice aged 2–8 months; controls were matched for age and sex with HLLKO mice. ▪, normal, early treatment (n = 5; 2–8 month-old mice); ▴, HLLKO, early treatment (n = 5; 2–8 months); ▾, normal, sham and late treatment (n = 5; 3–7 months); ♦, HLLKO, sham and late treatment (n = 5; 3–7 months). ** HLLKO early vs HLLKO late, p≤0.01.

Ten of 13 HLLKO mice (77%) entered a crisis after intraperitoneal KIC injection. Each of these 10 mice developed hyperammonemia >200 µmol/L (p<0.005 versus matched normal controls). Five hyperammonemic HLLKO mice received carglumate treatment one hour after hyperammonemia was first documented (Figure 7). The plasma ammonia level each HLLKO mouse decreased within two hours following carglumate (p<0.05) and in all 5 early-treated HLLKO mice, plasma ammonia was <200 µmol/L by 3 hours after carglumate treatment (not shown). In contrast, in a second group of 5 HLLKO mice that received a sham gavage instead of carglumate, plasma ammonia increased (p = 0.01) in comparison with treated mice (Figure 7). When these sham-treated HLLKO mice then received carglumate, four hours after the first documentation of hyperammonemia, plasma ammonia decreased to <200 µmol/L within two hours in 4/5 mice (p<0.05). The single late-treated non-responder, despite receiving a second dose of carglumate, showed a progressive increase in plasma ammonia and was sacrificed. Neither KIC nor carglumate administration affected plasma ammonia level in control mice.

### HLLKO mice have markedly abnormal mitochondrial ultrastructure

Histologically, control livers and livers from non-stressed HLLKO mice showed large droplets in some periportal hepatocytes and relative sparing of pericentral hepatocytes (Figure S1). In KIC-treated HLLKO liver, droplets tended to be medium-sized and be panlobular in distribution.

By electron microscopy (Figure S2), a high fraction of HLLKO hepatocyte mitochondria were abnormally dilated under nonstressed conditions, compared with normal controls. This was more marked in KIC loaded liver. In KIC-loaded HLLKO liver, dissolution of the mitochondrial matrix and sometimes vacuole formation with extinction of matricial systems was observed. Paracrystalline inclusions, such as described in disorders of the respiratory chain [23], were not seen.

### Peroxisome-related metabolites are normal in HLLKO mice

Plasma very long chain fatty acids, plasmalogens, cholesterol and sterol synthetic intermediates were similar in HLLKO and normal mice (not shown).

## Discussion

### HLLKO mice have many features of human inborn errors of acyl-CoA metabolism and show that liver is critical for the development of these signs

In mice, deficiency of HL confined to liver can produce the cardinal signs of classic inborn errors of acyl-CoA metabolism, namely systemic crises of hypoglycemia, hyperammonemia and coma. Caution must be exercised in drawing specific clinical conclusions from HLLKO mice because of the liver-specificity of HL deficiency of HLLKO mice and the many physiological differences between mice and humans. However, the finding of the same clinical signs as humans with the same enzyme deficiency strongly suggested that HLLKO mice would be useful for understanding the pathophysiology of HL deficiency in a general fashion.

### Acyl-CoA levels show characteristic patterns in HLLKO liver

In HLLKO liver, marked chronic differences occur in total and relative amounts of CoA esters, with major shifts in KIC-induced and spontaneous crises. During crises, HL-deficient liver shows reduced levels of acyl-CoAs such as acetyl-CoA that are essential for normal function and increased levels of leucine-related acyl-CoAs, which are potential inhibitors of acetyl-CoA or succinyl-CoA-dependent reactions.

In HL-deficient liver, leucine degradation is converted from a high capacity linear pathway to a network of interconnected pools. Enzyme reactions that normally are of little importance may become the principle route of leucine elimination in HLLKO liver, allowing carbon skeletons to trickle out at several places (Figure 5).

The relative levels of liver acyl-CoAs, plasma acylcarnitines and urinary free acids differ greatly at different steps of leucine degradation. Presumably this reflects the different properties of the enzymes at each metabolic step.

For example, excretion of large amounts of 3-methylglutaconic acid and of the related 3-methylglutaric acid is a major pathway for leucine carbon disposal in HL deficiency. In HLLKO liver, the high level of HMG-CoA is predicted to shift the equilibrium of 3-methylglutaconyl-CoA (3-MGO-CoA) hydratase towards 3-MGO-CoA, but the level of 3-MGO-CoA is very low. Of note, 3-MGO-CoA hydratase possesses hydrolase activity [24]. We speculate that hydrolysis maintains mitochondrial 3-MGO-CoA at a low level.

Extrahepatic tissues in HLLKO mice are predicted to have a normal capacity for leucine degradation, but this does not prevent the massive urinary excretion of leucine metabolites. This shows that the capacity for renal excretion is similar to or greater than that of nonhepatic tissues to capture and metabolise these compounds.

### Acyl-CoA-related mechanisms of hypoketotic hypoglycemia, hyperammonemia and mitochondrial swelling

**Figure 8:**
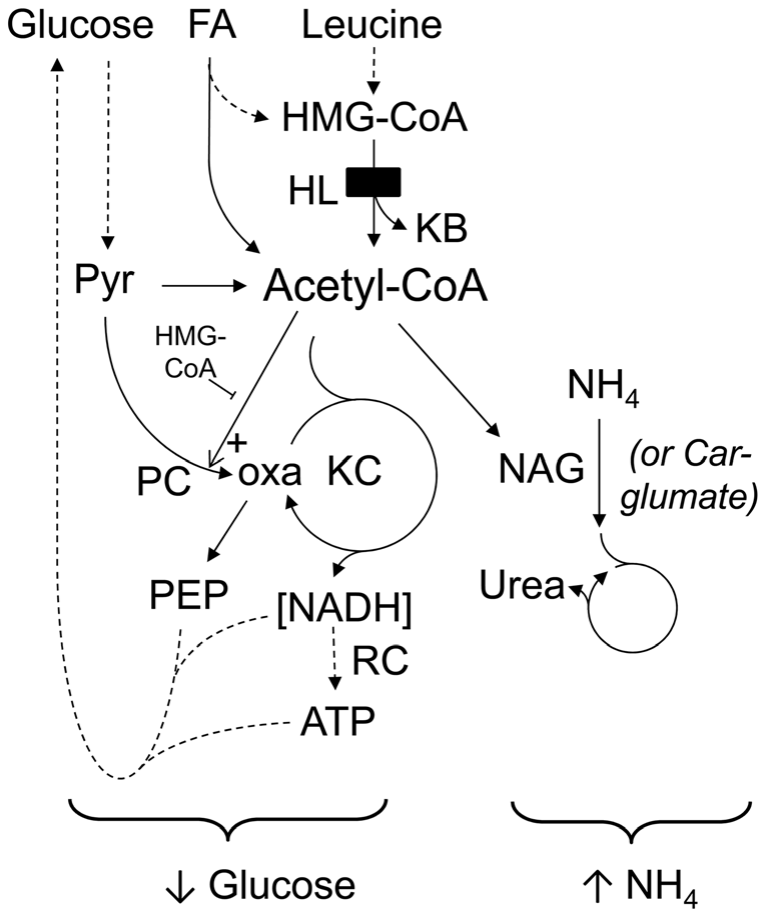
In HLLKO liver, acyl-CoAs play a crucial role in acute hypoglycemia and hyperammonemia. Acetyl-CoA is a critical point of convergence of glucose, fat and amino acid metabolism. Hypoglycemia can caused by at least two mechanisms related to low level of acetyl-CoA and abnormally high levels of other acyl-CoAs: lack of NADH and ATP production that normally power glucose synthesis, and lack of activation of pyruvate carboxylase (PC) [33]. Hyperammonemia can arise if a low level of acetyl-CoA will reduces the synthesis of N-acetylglutamate (NAG), which is essential for the urea cycle. Use of carglumate, a pharmacological substitute for NAG, reduces the hyperammonemia of acute crises. NADH and ATP-requiring steps of gluconeogenesis not discussed specifically are shown by dotted lines. Other abbreviations, KC, Krebs cycle; NADH, reduced nicotine adenine dinucleotide; oxa, oxaloacetate; PEP, phospho-*enol*-pyruvate; Pyr, pyruvate; RC, respiratory chain.

Hypoglycemia and hyperammonemia, two cardinal metabolic signs of acute crises, have strong potential links to acyl-CoA metabolism (Figure 8).

Pyruvate carboxylase (PC), the first enzyme of gluconeogenesis, is stimulated allosterically by acetyl-CoA [2], [25]. The Ka for activation of PC by acetyl-CoA is ∼140 µmol/L [9]. High levels of other acyl-CoAs can reduce this effect [25] (Figure 8). In this study, acyl-CoA levels were measured in whole tissues, allowing samples to be frozen within seconds after organ removal and increasing the accuracy of measurement by minimizing the risk of post-mortem changes in acyl-CoA levels. The calculated intramitochondrial concentration of HMG-CoA in HLLKO liver, ∼300 µmol/L, exceeds the reported Ki of 140 µmol/L for inhibition of acetyl-CoA stimulation of purified chicken liver PC [26]; this doubles the apparent Ka for acetyl-CoA stimulation of PC [27]. The effects of other leucine-related acyl-CoAs on acetyl-CoA-mediated stimulation of PC have not been tested directly but could further reduce the PC activity in HLLKO liver.

Inhibition of PC could explain the lack of increase of blood glucose following pyruvate loading in HLLKO mice. An alternative mechanism of hypoglycemia, which is compatible and synergistic with reduced PC, is reduced production of NADH and ATP. Both of these compounds are used in gluconeogenesis and the production of adequate amounts of each depends on normal Krebs cycle flux.

Inhibition of pyruvate carboxylase could reduce Krebs cycle flux, consistent with the decrease of pyruvate carboxylation observed in HLLKO mitochondria incubated with KIC. ^14^CO_2_ generation from [2-^14^C]pyruvate can also be reduced if the pool size of Krebs cycle intermediates is increased, if pyruvate transport into mitochondria is decreased, or if labelled carbon is diverted towards gluconeogenesis from pyruvate or exits the Krebs cycle at other points prior to producing CO_2_. Our results provide no evidence in favour of these mechanisms, and the pyruvate tolerance test gives strong evidence against increased pyruvate carboxylase activity. We cannot formally eliminate the other causes of reduced CO_2_ generation. However, acyl-CoA-mediated inhibition of pyruvate carboxylase is consistent with all of our data and provides a unifying explanation for both the lack of increase of blood glucose following pyruvate loading and for the reduction of ^14^CO_2_ generation from [2-^14^C]pyruvate in KIC-treated mitochondria from HLLKO mice.

In contrast to the high levels of leucine-related metabolites in HLLKO compared to normal liver, in HLLKO liver during crises, changes in free CoA, succinyl-CoA and acetyl-CoA were mild or absent. In KIC-loaded HLLKO liver, free CoA and succinyl-CoA showed trends to reduced levels compared with controls, but this did not reach significance. In contrast, intramitochondrial acetyl-CoA levels, calculated as described [9], were significantly reduced in HLLKO liver following KIC treatment (p<0.01, Figure 4), from ∼360 µmol/L to ∼190 µmol/L. In purified PC, this level of acetyl-CoA, in the presence of the constantly high level of HMG-CoA found in HLLKO liver, suffices to reduce PC activity [27], suggesting that reduction of acetyl-CoA may be the critical factor in precipitating hypoglycemia in HLLKO mice.

A chain of events can be traced from the primary metabolic defect to hyperammonemia (Figure 8): gene mutation and enzyme deficiency of HL, abnormal levels of biologically active acyl-CoAs and an acyl-CoA-dependent inhibition of N-acetylglutamate synthase, resulting in hyperammonemia. Of note, N-acetylglutamate synthase is an acetyl-CoA-dependent mitochondrial matrix enzyme that is essential for normal urea cycle function. Its Km for acetyl-CoA is 600 µmol/L [1], close to the measured physiological concentration of acetyl-CoA. Although HMG-CoA and related CoA esters were not specifically tested, N-acetylglutamate (NAG) synthase is known to be susceptible to inhibition by other CoA esters [1] and pharmacological replacement of the NAG synthase enzyme product by carglumate effectively reduces hyperammonemia in HLLKO mice in crises. The only known biological action of carglumate is as an analogue of N-acetylglutamate. Improvement of hyperammonemia following carglumate has also been reported in human inborn errors of acyl-CoA metabolism [1], [28]. The data strongly support an acyl-CoA-mediated mechanism for the hyperammonemia of HL deficiency and by extension, of other diseases of acyl-CoA metabolism.

Hypoglycemia, hyperammonemia and somnolence occur in many different diseases of acyl-CoA metabolism, each of which is predicted to have different abnormalities of acyl-CoA levels. The marked swelling of HLLKO liver mitochondria, particularly in samples obtained in KIC-induced crises, is similar to observations in methylmalonic acidemia, another disease of acyl-CoA metabolism for which ultrastructural data is available [29]. It will be interesting to test whether the acute crises of other inborn errors of acyl-CoA metabolism are characterized by acute reduction of acetyl-CoA and succinyl-CoA levels and by an increase in the concentration of other, potentially toxic CoAs. The properties of the other acyl-CoAs and of the metabolites which arise from them, accumulation of which is predicted to be different in each disease, may account for the clinical differences among these conditions.

### KIC-induced and spontaneous crises may arise by different pathways

HLLKO mice in spontaneous crises had evidence of increased fatty acid oxidation, including increases of dicarboxylic acids in urine (Table S1) and of long chain fatty acylcarnitines in plasma (Table S2). Spontaneous crises presumably develop over a longer period than the 1–4 hours required for the development of crises following acute KIC loading. This longer pre-crisis phase would permit activation of adipocyte lipolysis and liver fatty acid oxidation. Interestingly, in HL-deficient human patients, different sources of HMG-CoA have been implicated during infections, in which protein catabolism predominates, and fasting, in which non-protein sources like fatty acids play a larger role [30].

### Liver HL deficiency does not affect peroxisomal function tests

HL has targeting signals for mitochondria and for peroxisomes on its N- and C-termini, respectively and about 6% of mouse liver HL is peroxisomal [19]. The function of peroxisomal HL is unknown. In HLLKO mice, metabolites of peroxisomal beta oxidation and of the synthesis of plasmalogens and sterols were normal, showing that liver peroxisomal HL is not essential for these functions. Peroxisomes play major roles in lipid-rich tissues like brain, adrenal and testes. Since HLLKO mice only lack HL in hepatocytes, a role for peroxisomal HL cannot be eliminated in other tissues.

### HLLKO mice provide support for acyl-CoA-mediated pathogenesis

In inborn errors of acyl-CoA metabolism, the high concentrations of CoA esters are predicted to be the first step of the metabolic cascades from which all clinical manifestations arise. The acronym “CASTOR” has been applied to the state of coenzyme A sequestration, toxicity and/or redistribution predicted to occur in hereditary and acquired disorders of acyl-CoA metabolism [31]. HLLKO mice permitted the first direct, controlled study of the CASTOR hypothesis. They confirm central predictions of the CASTOR hypothesis in an inborn error of acyl-CoA metabolism: they demonstrate chronically abnormal levels of tissue acyl-CoAs, which intensify during stress, and provide the most complete evidence to date for a direct role of acyl-CoAs in the cardinal signs of hypoglycemia and hyperammonemia common to many inborn errors of acyl-CoA metabolism.

#### Note added in proof

During revision of this article, a publication describing another technique for tissue acyl-CoA profiling came to our attention [32].

## Supporting Information

Figure S1 **Liver histology of HLLKO and control mice.** (a) normal control; (b) HLLKO; (c) normal control 6 h after KIC injection and (d) HLLKO liver after KIC injection. (a–c) show moderate steatosis with large lipid droplets predominantly in a periportal location and relative sparing of pericentral hepatocytes. In (d), a panlobular distribution of medium-sized droplets is seen. All slides show hematoxylin and eosin staining, X200.
(TIF)

Figure S2 **HLLKO hepatocytes have marked abnormalities of mitochondrial ultrastructure.** (a) normal mitochondria in control hepatocytes following KIC injection. (b) HLLKO hepatocyte, nonstressed conditions, showing matrix swelling in some mitochondria. (c) HLLKO hepatocyte after KIC injection. Most mitochondria show severe hydropic swelling.
(TIF)

Table S1 **Selected urinary organic acids related to Krebs cycle, fatty acid and leucine metabolism.** Values shown are mean ± SEM; * p≤0.05; ** p≤0.01 compared to control; § p≤0.05; §§ p≤0.01 compared to HLLKO stable.
(RTF)

Table S2 **Plasma acylcarnitine levels in HLLKO mice and controls.** Values shown are mean ± SEM; * p≤0.05; ** p≤0.01 compared to control stable; § p≤0.05; §§ p≤0.01; §§§ p≤0.001 compared to HLLKO stable; † p≤0.05; †† p≤0.01 compared to control KIC.
(RTF)

Text S1 **Supporting materials and methods.**
(RTF)
