# Supplementary figures and images for: A Liver-Specific Defect of Acyl-CoA Degradation Produces Hyperammonemia, Hypoglycemia and a Distinct Hepatic Acyl-CoA Pattern
Source: PLoS One. 2013 Jul 5;8(7):e60581. doi: 10.1371/journal.pone.0060581 (PMC3702508; doi:10.1371/journal.pone.0060581)

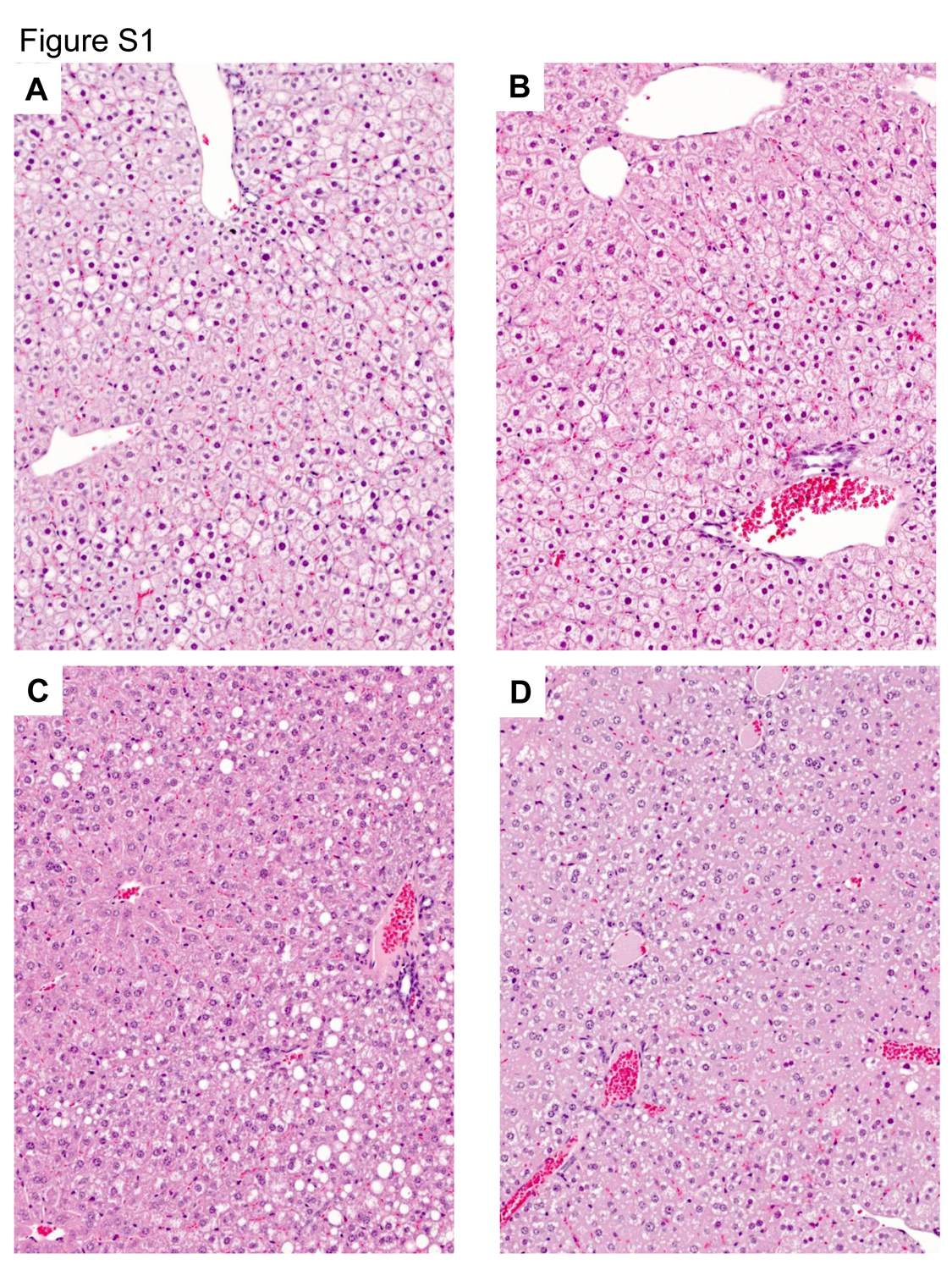

Supplement: Figure S1 — Liver histology of HLLKO and control mice. (a) normal control; (b) HLLKO; (c) normal control 6 h after KIC injection and (d) HLLKO liver after KIC injection. (a–c) show moderate steatosis with large lipid droplets predominantly in a periportal location and relative sparing of pericentral hepatocytes. In (d), a panlobular distribution of medium-sized droplets is seen. All slides show hematoxylin and eosin staining, X200. (TIF) [file pone.0060581.s001.tif]

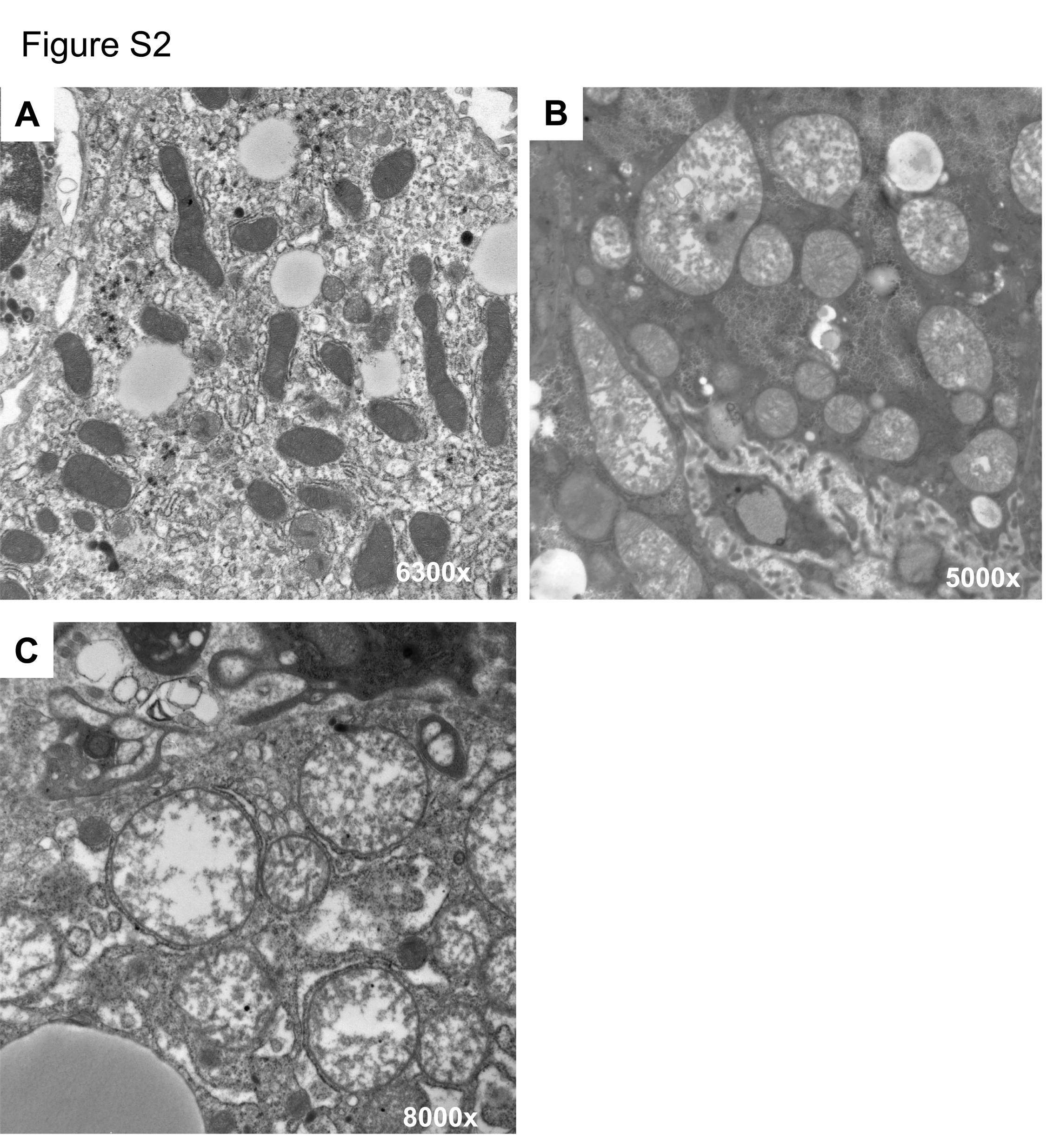

Supplement: Figure S2 — HLLKO hepatocytes have marked abnormalities of mitochondrial ultrastructure. (a) normal mitochondria in control hepatocytes following KIC injection. (b) HLLKO hepatocyte, nonstressed conditions, showing matrix swelling in some mitochondria. (c) HLLKO hepatocyte after KIC injection. Most mitochondria show severe hydropic swelling. (TIF) [file pone.0060581.s002.tif]
