# Supplementary material for: A Liver-Specific Defect of Acyl-CoA Degradation Produces Hyperammonemia, Hypoglycemia and a Distinct Hepatic Acyl-CoA Pattern
Source: PLoS One. 2013 Jul 5;8(7):e60581. doi: 10.1371/journal.pone.0060581 (PMC3702508; doi:10.1371/journal.pone.0060581)
Supplement: Table S1 — Selected urinary organic acids related to Krebs cycle, fatty acid and leucine metabolism. Values shown are mean ± SEM; * p≤0.05; ** p≤0.01 compared to control; § p≤0.05; §§ p≤0.01 compared to HLLKO stable. (RTF) [file pone.0060581.s003.rtf]

Table S1.  Selected urinary organic acids related to Krebs cycle, fatty acid and leucine metabolism. 
	CONTROL 	HLLKO STABLE	HLLKO CRISIS	
n	10	9	3	
Lactic	1620 ± 1010	1410± 1160	5960 ± 5740	
Citric	4660 ± 2750	5580 ± 1920	25570 ± 12650	
2-Ketoglutaric	468 ± 307	205 ± 150	1920 ± 1650	
Succinic	255 ± 81	385 ± 109	956 ± 274	
Fumaric	7.8 ± 4.1	13.1 ± 7.1	119 ± 57	
Malic	37.0 ± 22.2	111 ± 43	808 ± 419	
Ethylmalonic	12.4 ± 3.2	7.6 ± 1.7	33.7 ± 2.7**§§	
Methylsuccinic	11.8 ± 2.2	7.1 ± 1.9	19.0 ± 5.5	
Adipic	33.8 ± 9.7	25.4 ± 22.3	625 ± 186	
Suberic	1.1 ± 0.5	3.6 ± 3.6	116 ± 40.5	
Sebacic	0.0 ± 0.0	0.1 ± 0.1	30.7 ± 6.4*§	
3-Hydroxysebacic	0.3 ± 0.2	1.7 ± 1.6	132 ± 102	
Hexanoylglycine	5.7 ± 1.4	5.9 ± 1.1	12.7 ± 4.3	
3-Hydroxyisovaleric	3.8 ± 2.2	2.2 ± 1.6	584 ± 229	
Isovalerylglycine	20.2 ± 7.2	30.3 ± 13.2	8.7 ± 2.33	
3-Methylglutaconic	40.4 ± 27.1	717 ± 237*	37900 ± 701*§	
3-Methylglutaric	0.7 ± 0.7	19.3 ± 6.3*	68.0 ± 31.6	
3-Methylcrotonylglycine	0.2 ± 0.2	21.2 ± 7.2*	131.0 ± 49.5	
3-Hydroxy-3-Methylglutaric	20.9 ± 10.4	294 ± 197	6540 ± 3790	
4-Hydroxy-phenylpyruvic	11.7 ± 8.5	8.9 ± 4.9	21.7 ± 10.9	
4-Hydroxyphenyllactic	5.8 ± 1.2	10.8 ± 2.4*	10.7 ± 0.7	
        
Legend : Values shown are mean ± SEM; * p≤0.05 ; ** p≤0.01 compared to control ; § p≤0.05; §§ p≤0.01 compared to HLLKO stable
