# Supplementary material for: A Liver-Specific Defect of Acyl-CoA Degradation Produces Hyperammonemia, Hypoglycemia and a Distinct Hepatic Acyl-CoA Pattern
Source: PLoS One. 2013 Jul 5;8(7):e60581. doi: 10.1371/journal.pone.0060581 (PMC3702508; doi:10.1371/journal.pone.0060581)
Supplement: Table S2 — Plasma acylcarnitine levels in HLLKO mice and controls. Values shown are mean ± SEM; * p≤0.05; ** p≤0.01 compared to control stable; § p≤0.05; §§ p≤0.01; §§§ p≤0.001 compared to HLLKO stable; † p≤0.05; †† p≤0.01 compared to control KIC. (RTF) [file pone.0060581.s004.rtf]

Table S2.  Plasma acylcarnitine levels in HLLKO mice and controls.  
Category	CONTROL STABLE	CONTROL KIC	HLLKO STABLE	HLLKO KIC	HLLKO CRISIS	
n	14	3	12	3	5	
Free Carnitine	  23.8 ± 1.9	  9.97 ± 8.49	  19.8 ± 2.2	  15.0 ± 3.0	  19.2 ± 3.8	
C-2 (Acetyl)	  12.9 ± 1.1	  20.0 ± 1.3**	  11.3 ± 1.2††	  14.6 ± 4.8	  19.3 ± 4.1	
C-3 (Propionyl)	0.210 ± 0.022	0.243 ± 0.058	0.258 ± 0.034	0.127 ± 0.048	0.130 ± 0.024*§§	
C-4	0.433 ± 0.040	0.383 ± 0.019	0.472 ± 0.116	0.247 ± 0.094	0.168 ± 0.066*§†	
C-5-Hydroxy	0.021 ± 0.002	0.093 ± 0.007**	0.026 ± 0.003††	0.103 ± 0.015§	0.102 ± 0.019*§	
C-8	0.014 ± 0.002	0.020 ± 0.000*	0.014 ± 0.002††	0.017 ± 0.007	0.026 ± 0.007	
C-10:2	0.023 ± 0.003	0.030 ± 0.006	0.015 ± 0.005	0.037 ± 0.022	0.050 ± 0.025	
C-10:1	0.018 ± 0.002	0.033 ± 0.009	0.014 ± 0.003	0.023 ± 0.013	0.034 ± 0.014	
C-10	0.020 ± 0.002	0.017 ± 0.007	0.015 ± 0.002*	0.023 ± 0.003	0.086 ± 0.017*§†	
C-4-Dicarboxylic	0.019 ± 0.003	0.053 ± 0.020	0.025 ± 0.004	0.043 ± 0.019	0.032 ± 0.015	
C-5-Dicarboxylic	0.010 ± 0.001	0.017 ± 0.003	0.018 ± 0.003*	0.033 ± 0.019	0.074 ± 0.019§†	
C-12:1	0.009 ± 0.002	0.023 ± 0.009	0.005 ± 0.002	0.037 ± 0.007§	0.066 ± 0.029	
C-12	0.021 ± 0.003	0.037 ± 0.009	0.017 ± 0.002	0.057 ± 0.007§	0.292 ± 0.083*§†	
C-6-Dicarboxylic	0.011 ± 0.001	0.013 ± 0.003	0.015 ± 0.002	0.030 ± 0.010	0.036 ± 0.022	
C-14:2	0.024 ± 0.003	0.337 ± 0.253	0.016 ± 0.002*	0.157 ± 0.132	0.132 ± 0.049	
C-14:1	0.062 ± 0.015	0.073 ± 0.015	0.041 ± 0.008	0.180 ± 0.045	0.314 ± 0.065*§†	
C-14	0.064 ± 0.010	0.063 ± 0.019	0.046 ± 0.007	0.247 ± 0.015§§††	0.916 ± 0.190*§†	
C-14-Hydroxy	0.004 ± 0.001	0.003 ± 0.003	0.001 ± 0.001	0.010 ± 0.000§§§	0.048 ± 0.014*§†	
C-16:1	0.061 ± 0.013	0.063 ± 0.007	0.047 ± 0.011	0.660 ± 0.152	1.050 ± 0.229*§†	
C-16	0.121 ± 0.018	0.127 ± 0.052	0.110 ± 0.017	0.870 ± 0.294	3.276 ± 0.528**§§††	
C-16:1-Hydroxy	0.009 ± 0.001	0.010 ± 0.000	0.007 ± 0.001	0.030 ± 0.000§§§	0.104 ± 0.025*§†	
C-16-Hydroxy	0.010 ± 0.000	0.020 ± 0.006	0.010 ± 0.000	0.020 ± 0.006	0.114 ± 0.033*§†	
C-18:2	0.033 ± 0.005	0.053 ± 0.023	0.024 ± 0.004	0.153 ± 0.038	0.994 ± 0.358	
C-18:1	0.231 ± 0.048	0.137 ± 0.019*	0.157 ± 0.030	1.23 ± 0.23§†	2.75 ± 0.65*§†	
C-18	0.044 ± 0.007	0.037 ± 0.017	0.052 ± 0.005	0.133 ± 0.043	0.242 ± 0.047*§	
C-18:2-Hydroxy	0.013 ± 0.003	0.010 ± 0.000	0.008 ± 0.001	0.017 ± 0.003	0.082 ± 0.018*§†	
C-18:1-Hydroxy	0.015 ± 0.003	0.010 ± 0.000*	0.010 ± 0.002	0.040 ± 0.006§†	0.100 ± 0.029§†	
C-18-Hydroxy	0.014 ± 0.001	0.010 ± 0.000**	0.011 ± 0.002	0.023 ± 0.009	0.028 ± 0.007	
Legend : Values shown are mean ± SEM; * p≤0.05 ; ** p≤0.01 compared to control stable ; § p≤0.05; §§ p≤0.01; §§§ p≤0.001 compared to HLLKO stable ; † p≤0.05 ; †† p≤0.01 compared to control KIC.
