# Supplementary material for: A Liver-Specific Defect of Acyl-CoA Degradation Produces Hyperammonemia, Hypoglycemia and a Distinct Hepatic Acyl-CoA Pattern
Source: PLoS One. 2013 Jul 5;8(7):e60581. doi: 10.1371/journal.pone.0060581 (PMC3702508; doi:10.1371/journal.pone.0060581)
Supplement: Text S1 — Supporting materials and methods. (RTF) [file pone.0060581.s005.rtf]

SUPPORTING METHODS
A liver-specific defect of acyl-CoA degradation produces hyperammonemia, hypoglycemia and a distinct hepatic acyl-CoA pattern.  

Nicolas Gauthier 1,2, Jiang Wei Wu 1, Shu Pei Wang 1, Pierre Allard 1, Orval A Mamer 3, Lawrence Sweetman 4, Ann B. Moser 5, Lisa Kratz 5, Fernando Alvarez1 , Yves Robitaille1, François Lépine 6 and Grant A Mitchell 1,2.  
1Centre de Recherche and Département de Pédiatrie (NG, JWW, SPW, PA, FA, GAM) or Département de Pathologie (YR), CHU Sainte-Justine, Université de Montréal, Montréal, Canada
2Département de Biochimie, Université de Montréal, Montréal, Canada
3Goodman Cancer Research Centre, McGill University, Montréal, Canada
4Institute of Metabolic Disease, Baylor Research Institute, Dallas, TX, USA
5 The Hugo W Moser Research Institute, The Kennedy-Krieger Institute, Johns Hopkins School of Medicine, Baltimore, Maryland
6INRS-Institut Armand-Frappier, Laval, Canada
* Running title: Acyl-CoAs and Liver Disease in Mice


SUPPLEMENTAL Experimental procedures

Targeting Vector Construction.  For the conditional targeting vector for HL, we chose to excise exon 2 of the mouse Hmgcl gene.  This exon contains several catalytically-essential residues [1]. To create the insertion vector shown in Figure 1a, a DNA fragment containing the neomycin resistance gene and 129Sv/J mouse HL exon 2 flanked by Lox sites, was inserted into a 129 Sv/J genomic clone. A thymidine kinase gene with a phosphoglycerate kinase promoter (not shown in the figure) was inserted upstream of the short arm.

Hmgcl gene targeting and genotyping of the targeted HL allele  Targeting of Hmgcl was performed in J1 embryonic stem cells as described [2].  Neomycin-resistant, thymidine-sensitive colonies were studied. Cells were washed with phosphate buffered saline. Lysates were digested with proteinase K, and then heated at 95°C for 6 min to inactivate Proteinase K.  20% of the lysate was used directly for PCR amplification.  The sense primer HLM28 (5'-CCGTTCGCTTCAAATCAGGC–3'), which is outside of the targeting vector, corresponds to residues -1054 to -1034 nt 5' of exon 2.  The sequences of antisense primer Neo-I and PCR conditions were as described previously [3]. The diagnostic 1.3 kb amplicon indicated the targeted allele.  It was present in three of 96 clones (3.2%). The targeted ES cell clones were confirmed by Southern blotting as follows.  ES cell genomic DNA (5 µg) was digested with both HindIII and Xba I and probed with an amplified 330 nt fragment located outside the targeting vector and corresponding to residues 799-1130 nt upstream from the acceptor splice site of exon 2. The probe detects 3.8 kb (normal) and 4.9 kb (targeted) fragments.  Southern blotting was performed as described previously [3].

Genotyping of HLL and Cre+ alleles  PCR reactions for genotyping normal (+/+), targeted (L/L) and heterozygous (+/L) mice were performed with 3 primers: HLM 65 (sense primer, 5'-GAGCACAGCTTCCTTGCTTA-3', corresponding to residues -160 to -180 nt upstream of exon 2), HLM 66 (antisense primer, 5'-ACAGTCTTGAGCCACTTGATAT-3', residues +145 to +165 nt downstream of exon 2) and the antisense primer Neo1 [3] PCR conditions were described [3] except that cycles were as follows : 94°C, 45 sec; 55°C, 45 sec; 72°C, 30 sec for 35 cycles with a final 10 min at 72°C then 4°C. 
	Amplification to detect the Cre transgene was carried out with Cre-1 (5'-GATGGACATGTTCAGGGATC-3' a sense primer spanning Cre cDNA positions 80 to 100-nt with respect to the A residue of the initiation ATG codon) and Cre-2 (5'-AGCTTGCATGATCTCCGGTA-3', an antisense primer spanning Cre cDNA residues 916 to 936). Cycles were as follows : 94°C, 45 sec; 60°C, 45 sec; 72°C, 1 m 30 sec for 30 cycles with a final 10 min at 72°C then 4°C.

SUPPLEMENTAL REFERENCES

1. Mitchell GA, Ozand PT, Robert MF, Ashmarina L, Roberts J, et al. (1998) HMG CoA lyase deficiency: identification of five causal point mutations in codons 41 and 42, including a frequent Saudi Arabian mutation, R41Q. Am J Hum Genet 62: 295-300.
2. Sirois J, Cote JF, Charest A, Uetani N, Bourdeau A, et al. (2006) Essential function of PTP-PEST during mouse embryonic vascularization, mesenchyme formation, neurogenesis and early liver development. Mech Dev 123: 869-880.
3. Wang SP, Marth JD, Oligny LL, Vachon M, Robert MF, et al. (1998) 3-Hydroxy-3-methylglutaryl-CoA lyase (HL): gene targeting causes prenatal lethality in HL-deficient mice. Hum Mol Genet 7: 2057-2062.
